# Renormalization techniques for inflation systems and some of their applications

**DOI:** 10.1107/S2053273326003918

**Published:** 2026-05-26

**Authors:** Michael Baake, Franz Gähler, Anna Klick, Neil Mañibo, Jan Mazáč

**Affiliations:** a Fakultät für Mathematik Bielefeld University Postfach 100131 33501 Bielefeld Germany; Université de Lorraine, France

**Keywords:** renormalization, inflation tilings

## Abstract

Exact renormalization techniques are important and powerful, particularly for inflation-generated systems. We review recent results in this direction. We recall the necessary notions for inflation systems and show the renormalization principle, which allows us to obtain exact values of highly erratic functions, such as window covariograms. We apply these techniques to compute the diffraction pattern of the new monotile tilings with arbitrary precision. We also recall a recent invariant for a system with pure-point spectrum, the orbit separation dimension, and its relation to renormalization. Lastly, we recall results beyond the pure-point spectrum setting and show how renormalization and Lyapunov exponents can be used to exclude the presence of absolutely continuous parts of the spectra.

## Abstract 2

In this work, renormalization methods for quantities related to the diffraction of inflation systems are surveyed.

## 1. Introduction and preliminaries

The theory of aperiodic order is a mathematical discipline that emerged from the discovery of quasicrystals. It is concerned with the rigorous analysis of spatial structures without periodicity, but prevalent long-range positional and orientational order. In view of important applications in crystallography and materials science, one often works with tilings or Delone sets. Of particular relevance is the frequent presence of hierarchical structures, which is often related to an underlying inflation rule, or the compatibility with one. All the usual suspects in two and three dimensions, such as the Penrose and Ammann–Beenker tilings (2D), or the Ammann–Kramer and Danzer tilings (3D), have such a hierarchical structure. Such an inflation rule gives rise to exact renormalization schemes for many objects, and it is the goal of this survey to demonstrate the power of such a structure for quantities of practical relevance. In particular, we cover correlations, covariograms, diffraction, topological invariants and spectra, where we employ a uniform approach via the combination of inflation tilings with the model set description of characteristic point sets. We keep our exposition at an informal level, and refer to the relevant literature for details and proofs.

Tilings are well studied objects in the theory of aperiodic order, which have a lot of connections to physics. A tiling can be created in various ways, for instance via inflation rules. These prescribe how to replace an inflated version of a particular type of tile (a prototile) with a collection of other tiles. The notion of an inflation rule is quite intuitive and self-explanatory, which we summarize as follows.

Definition 1.1 Let 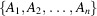 be a set of topologically regular, closed prototiles with almost no boundary in 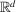 and with finite volume. An *inflation rule* with inflation multiplier 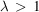 is a collection of mappings 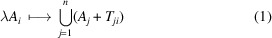 satisfying the following conditions:

(i) 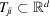 are all finite, and 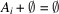.

(ii) The sets on the right-hand side of equation (1) have pairwise disjoint interiors.

(iii) The image of two tiles with disjoint interiors consists of tiles with disjoint interiors.

(iv) 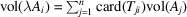 holds for all 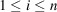.

Note that one can *always* change the shape of the prototiles (and create new ones with a usually fractal shape called *fractiles*) and turn the inflation rule (1) into an exact equation. In such a case, we speak of a *stone inflation*.

The sets of displacements 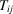 will play a crucial role in what follows. Therefore, we collect them into a set-valued matrix 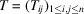, called the *displacement matrix*. It appears in the literature under various names. In the case of substitution tilings with integer scaling factors, it is usually called a *digit set matrix* (Lagarias & Wang, 1996 ▸; Vince, 2000 ▸). The matrix 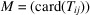 is called the *inflation matrix* and takes the role of the substitution matrix. We require the inflation matrix to be primitive, which ensures that all corresponding tilings look the same locally, independent of the legal seed with which one starts (Baake & Grimm, 2013 ▸).

Now, in every prototile, we can select a suitable reference point, called *control point*, and the entire tiling 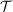 can then be described as a collection of translates of prototiles. Formally, 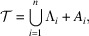
and the sets 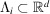 of control points inherit the inflation structure from equation (1). This induces the equations 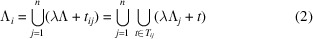
for 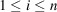, where all unions are disjoint.

An inflation tiling can only have non-trivial point spectrum (Bragg peaks) if its inflation factor is a Pisot–Vijayaraghavan number (PV number) (Solomyak, 1997 ▸, 1999 ▸), which is the root of an irreducible polynomial with integer coefficients, all of whose other roots are strictly smaller than 1 in modulus. The same condition is also necessary for an inflation tiling to be a cut-and-project tiling [or mutually locally derivable (MLD) with one]. Conversely, in one dimension, the *Pisot substitution conjecture* (Akiyama *et al.*, 2015 ▸) states that to have pure-point spectrum (pure Bragg diffraction), it is sufficient to have a PV number as inflation factor and an inflation matrix with an irreducible characteristic polynomial. For other cases (more than one dimension, reducible characteristic polynomial), there are methods to check whether the spectrum is pure point (Lee *et al.*, 2002 ▸). If this is the case, the set of control points 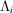 can be shown to arise from a *cut-and-project scheme* (CPS)
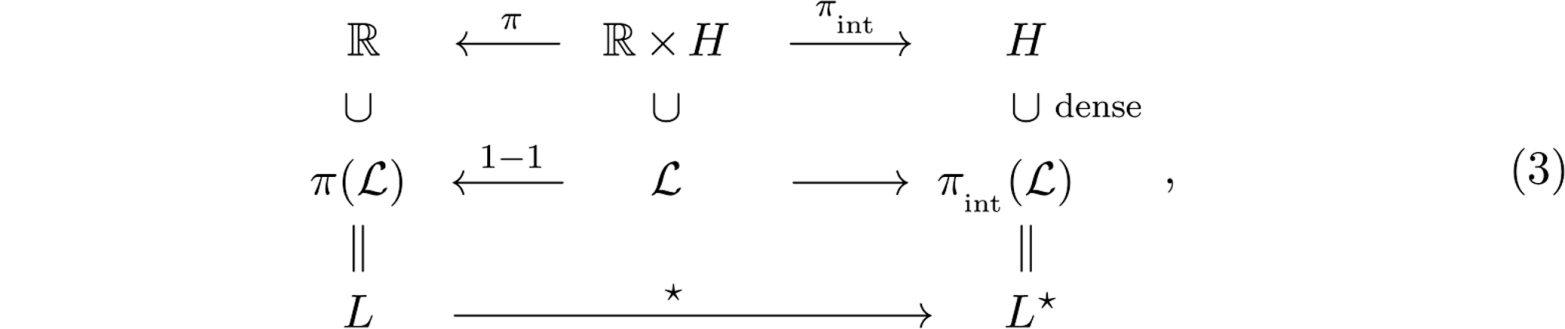
where 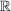 is the *physical space*, a locally compact Abelian group (LCAG) *H* is the *internal space*, and 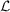 stands for a lattice (a co-compact, discrete subgroup) in 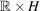. We also have a pair of natural projections 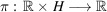, 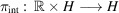 such that 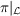 is injective and 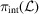 is dense in *H*. If 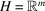, we call the CPS *Euclidean*. The injectivity condition ensures that 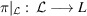 is a bijection. Therefore, one can define the *star map*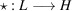 for any 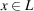 as 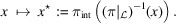
For the CPS from above, we define a *model set* as 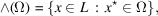
where a non-empty compact subset 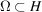 is known as its *window*. Whenever the Pisot substitution conjecture holds, we are guaranteed that there exist windows 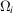 such that 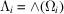, up to a set of zero density, and the windows are solutions to an iterated function system. Indeed, set 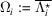. Then, taking the 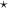-image and closure of equation (2) yields the desired iterated function system (IFS)
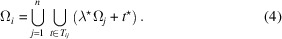
The same type of embedding can be obtained whenever the inflation factor is a PV number, also when a pure-point spectrum is not guaranteed *a priori*, for instance, for tilings in 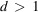 dimensions. We can still find a solution to (4), estimate its volume, and compare the densities of the sets 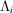 and 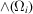. If these densities agree for all *i*, the sets 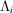, which are always contained in 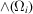, are given by the cut-and-project scheme (up to a set of density zero), so that indeed we have a system with pure-point spectrum.

For more details and further references and background, see Baake & Grimm (2013 ▸), Mazáč (2025*a* ▸), Sing (2007 ▸).

## 2. Covariograms

The Pisot substitution conjecture holds for unimodular, primitive Pisot substitutions with an irreducible characteristic polynomial on a two-letter alphabet (Hollander & Solomyak, 2003 ▸). This permits a model set description with one-dimensional internal space 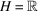 in the case of unimodular substitutions. In general (unless the windows are finite unions of intervals), the solutions of (4) are known as *Rauzy fractals* (Siegel & Thuswaldner, 2009 ▸), but in this particular one-dimensional setting, they belong to a special class called *Cantorvals*.

Definition 2.1 A *Cantorval* is a compact subset of 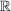 with uncountably many connected components, none of which are isolated, and it is equal to the closure of its interior. An *M*-*Cantorval* (or *symmetric* Cantorval) is a Cantorval with the additional property that the boundary of its interior is a Cantor set.

As we will always deal with symmetric Cantorvals, we drop the adjective ‘symmetric’ and refer to them simply as Cantorvals. These sets have a complicated structure, yet they define a regular model set, as they have positive Lebesgue measure, they are closures of their interiors and thus contain no isolated points, and have non-intersecting interiors and ‘almost no boundary’ (Sing, 2007 ▸, Cor. 6.66).

In general, one may ask when such a substitution gives rise to a Cantorval window. For example, the classic Fibonacci substitution can be described as a model set with an interval as its window. Nevertheless, the interval is still a solution to an IFS of the form (4) [see Baake & Grimm (2013 ▸), Klick (2024 ▸) for details]. For the class of unimodular primitive Pisot substitutions, we have the following simple criterion.

Theorem 2.2 (Baake et al., 2024, Thm. 4.1) Let ϱ be a primitive unimodular Pisot substitution on a binary alphabet with a model set realization with windows 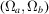. Denote 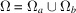. If the boundary of the window, 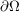, has positive Hausdorff dimension, then 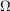, 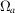, and 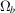 are Cantorvals.

Moreover, if we impose some mild additional restrictions on the substitutions, we obtain their classification based on the windows (see Mazáč, 2025*a* ▸, Cor. 2.4.6). We refer the reader to Baake *et al.* (2024 ▸), Mazáč (2025*a* ▸) for a more detailed discussion on Cantorvals and their role in the theory of aperiodic order.

Now that we know when we have a Cantorval or not, the next question for us is how this influences the structure of its *covariogram*.

Definition 2.3 Let 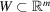 be a non-empty compact set. The *covariogram* of *W* is 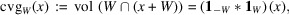
where 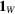 is the characteristic function of *W*, and 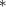 stands for the usual convolution of two functions 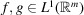 given by 
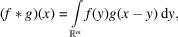
with the usual understanding that this is well defined for almost all 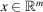.

Such functions play a key role in the diffraction of model sets. First of all, a covariogram describes certain patch frequencies in our system, and it determines the autocorrelation measure of the system (up to a scaling). Next, after taking the Fourier transform (and yes, there is a rigorous way of doing this, as will be explained in the next section), the covariogram provides the intensities of the Bragg peaks.

Now, let us consider the relative frequency of a control point of type *i* and a control point of type *j* (to the right of *i*) at a distance 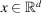, which is given by 
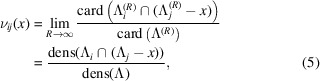
where we use the notation 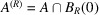 for any set 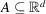 and any 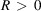. If 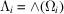, Moody’s uniform distribution theorem (Moody, 2002 ▸) for model sets implies 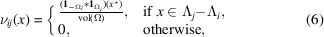
which gives the desired connection between covariograms and relative frequencies (or pair correlations). The inflation structure of the underlying structure (and suitable ergodic properties) permits the use of renormalization techniques for the pair correlations as follows.

Theorem 2.4 (Mañibo, 2019, Prop. 2.2.1) Let 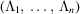 be a fixed point of a primitive geometric inflation ϱ with inflation factor 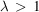 arising from a substitution over an *n*-letter alphabet 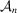. Then, the pair correlations 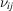 exist uniformly on the hull 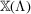, and satisfy the *exact* renormalization relations 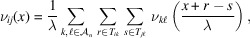
where 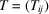 is the displacement matrix of the inflation ϱ. We note that this theorem can be extended, and using the same approach, one obtains all possible patch frequencies [see Mazáč (2025*b* ▸) for details].

**Figure 1:**
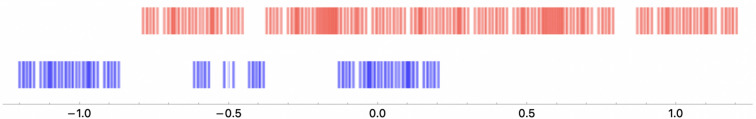
The window of the tiling corresponding to ϱ from (7); 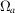 is red (top) and 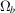 is blue (bottom). The windows are one-dimensional, but we assign some fixed arbitrary height to the points for illustration. The windows are measure-theoretically disjoint, but the resolution is limited by the large Hausdorff dimension of the window boundaries.

Using Theorem 2.4[Statement theorem2.4], we can compute and visualize the window covariogram, as we demonstrate with an example. Consider the substitution
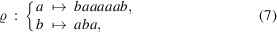
which we abbreviate as 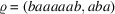. Using standard techniques, we can derive its self-similar version. Since the substitution is Pisot, it possesses a model set description, with the window system shown in Fig. 1 ▸. It has a fractal boundary whose Hausdorff dimension is 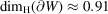; see Mazáč (2025*b* ▸), Sing (2007 ▸), Siegel & Thuswaldner (2009 ▸) for how to compute this quantity. The critical point is that calculating the covariogram via its definition as window overlaps for such a fractal is impossible. Thus, we turn to the renormalization procedure, as described in Theorem 2.4[Statement theorem2.4], giving the following relations.

Proposition 2.5 The pair correlations 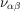 with 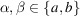 of the tiling corresponding to ϱ satisfy the exact renormalization relations:
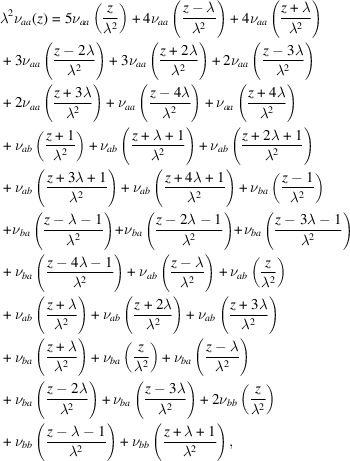

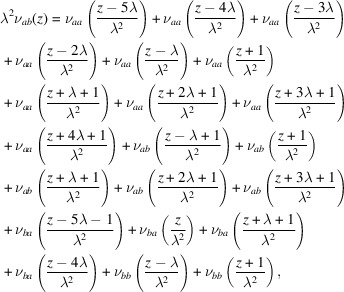

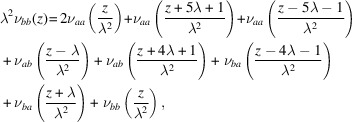
together with 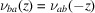, 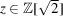, where 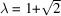 and 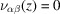 for 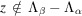.

The proof of this proposition is entirely analogous to other examples included in Baake & Gähler (2016 ▸), Baake *et al.* (2019*b* ▸), Baake *et al.* (2025*e* ▸), Klick (2024 ▸), and thus omitted. Now, this is an infinite set of linear equations. However, via the inflation structure, all arguments with 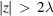 are recursively determined from the *self-consistent* part of the equations, which is given in Table 1 ▸; see Baake *et al.* (2019*b* ▸) for details and for a proof of the uniqueness of the solution.

**Figure 2:**
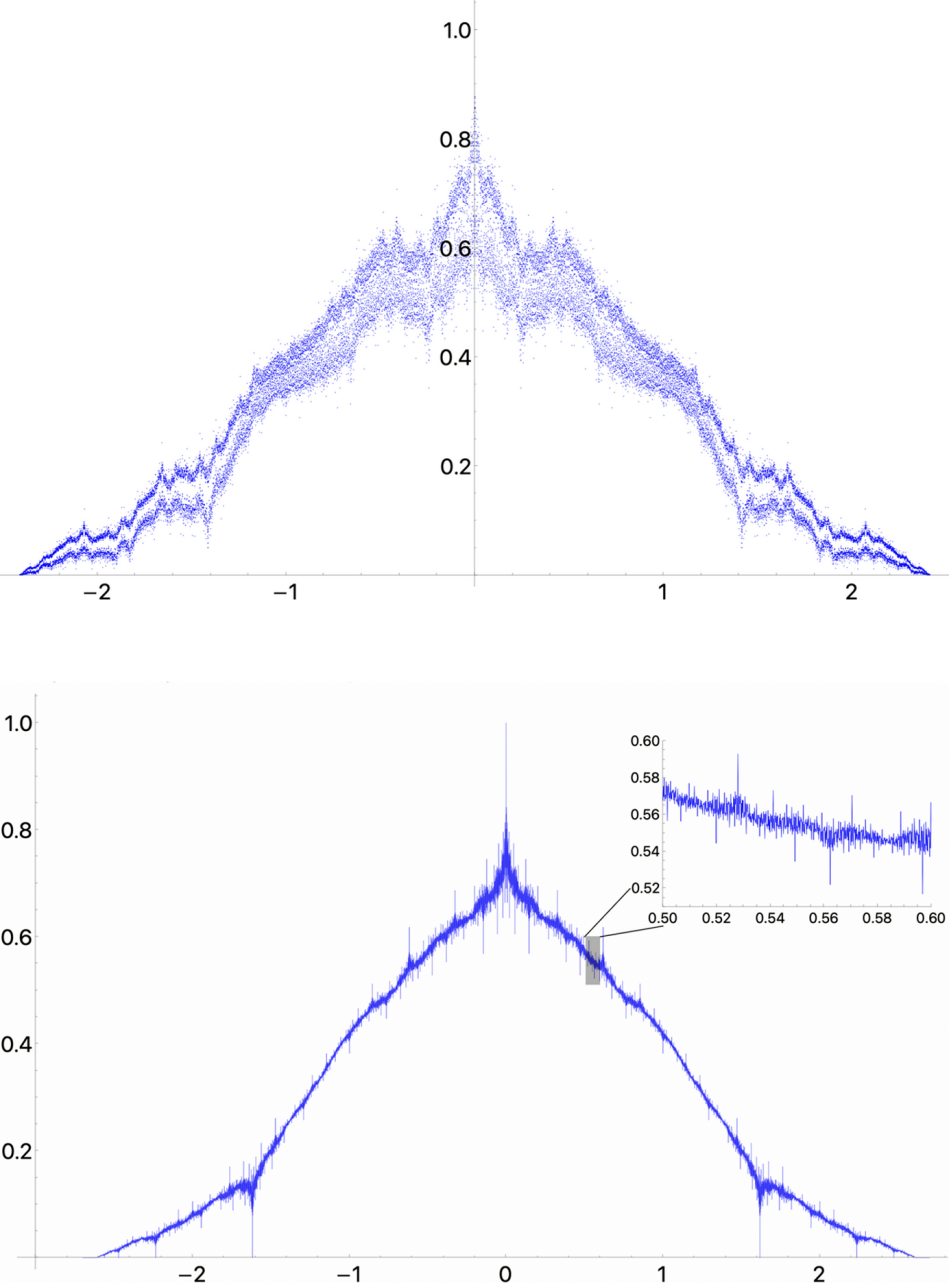
Upper: point plot, with 33877 points, of the covariogram of the window of Fig. 1 ▸. Lower: plot with 43205 points of the window corresponding to the reshuffled Fibonacci substitution [see Baake *et al.* (2025*e* ▸) for details]. A small inset is included to demonstrate the highly irregular behaviour.

With this self-consistent part in hand, serving as our seed, we can use the recursive nature of the renormalization relations to *exactly* calculate arbitrary values of the covariogram at points from 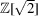, which is a dense subset of 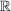. The plot of the covariogram is shown in Fig. 2 ▸. We see that the function seems to have a highly discontinuous nature, yet indeed is a continuous function, as it is the convolution of two functions that are both 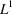 and 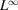 (Klick, 2024 ▸).

**Figure 3:**
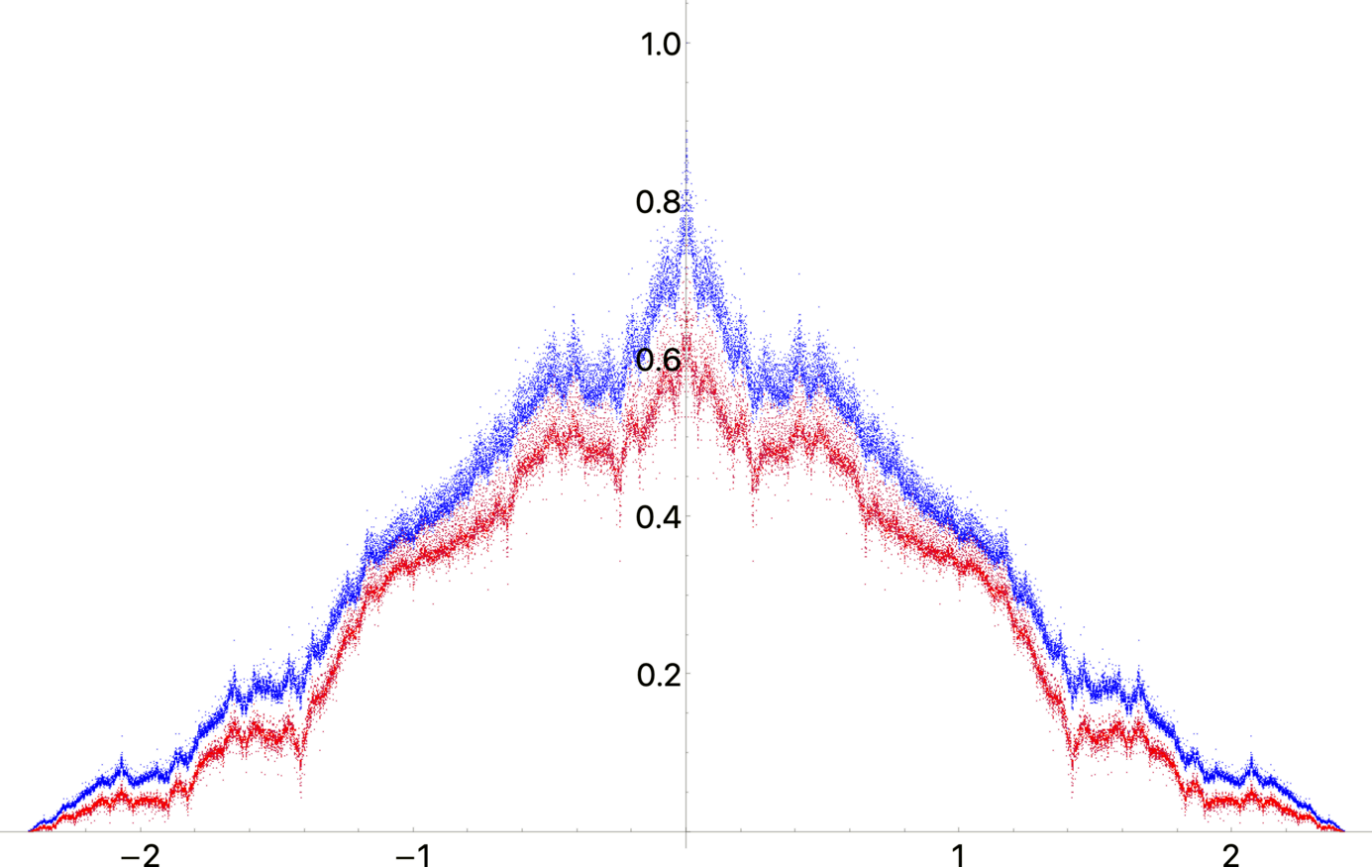
Point plot, with 33877 points, of the covariogram of the window of Fig. 1 ▸. Here, the splitting behaviour is highlighted: the distances 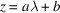 involving an even (odd) number of *b*’s are blue (red).

Moreover, this plot is an accurate representation of the function, due to the 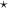-images of points from the physical space being both uniformly distributed and dense in the window (Baake & Grimm, 2013 ▸), combined with the continuity of the function. To account for this behaviour, a first reaction may be to attribute it to the high dimension of the window boundary; after all, this would naturally make the window overlap volume quite irregular. This fails to be true, as the second plot in Fig. 2 ▸, the reshuffled Fibonacci substitution 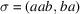, comes from a window with an even higher boundary dimension of approximately 0.92. Rather, this split, as shown in Fig. 3 ▸, is an artefact of the combinatorial structure of the substitution (*i.e.* from the number of *b* tiles at distance 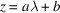), which can be seen on the level of the renormalization relations, but is outside the scope of this work (but is well worth further examination). There is no contradiction here, as the figure shows only an approximation of a continuous function. By adding more points to the plot and increasing the resolution, the gap will slowly close.

## 3. Diffraction of aperiodic monotiles

About two years ago, two families of tilings of the plane were discovered, both using a single non-convex polygon as the tile, the so-called Hat tiling (Smith *et al.*, 2024*a* ▸) and the Spectre tiling (Smith *et al.*, 2024*b* ▸). They both provide a partial solution to the monotile problem, as they both admit only aperiodic tilings.

The Hat tiling consists of 12 different prototiles with respect to translations: six Hats, which differ by a rotation by 60°, and six flipped versions of Hats – the anti-Hats, which also come in six different orientations. The Spectre tiling does not need the reflected version of the prototile; however, the Spectre tile appears in 12 different orientations (even though the tiling has only sixfold symmetry, as we shall see later). The 12 prototiles can be divided into two classes, Spectres versus shadow Spectres, with each tile related by a 60° rotation, and both classes related by a 30° rotation.

For the Hat tiling as well as the Spectre tiling, there is a class of prototiles that is dominant in the tiling, meaning that the relative frequency of, say, Hats is 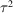-times higher than the relative frequency of anti-Hats. Here, τ stands for the golden ratio. The same phenomenon, with a different irrational factor, happens for Spectres versus shadow Spectres.

This small observation stands behind the reason why both tilings are considered to be only a partial solution to the monotile problem – both tilings consist of two locally indistinguishable (LI) classes. In other words, if one places a single tile on the plane, there are precisely two types of ways to finish the tiling, and these two resulting tiling classes do not differ by a translation. In the case of the Hat tiling, they are mirror images of each other, and in the Spectre case, they are related by a 30° rotation.

The long-range order of both tilings was determined, and it turns out that both tilings are quasiperiodic in the sense of Weyl (Baake *et al.*, 2025*c* ▸; Baake *et al.*, 2025*d* ▸). Indeed, it was also shown that both can be obtained as a reprojection of a model set, meaning that, for each tiling, there exists a four-dimensional Euclidean cut-and-project scheme and a window such that a non-canonical projection of the points from the strip determined by the window gives the control points of the desired tiling. These observations also imply that both tilings are pure-point diffractive (Baake *et al.*, 2025*b* ▸).

To derive the diffraction, *i.e.* the positions of the Bragg peaks and their intensities, one can use the renormalization techniques as well (in fact, it seems to be inevitable for these tilings). To do so, one has to find a particular tiling within the Hat/Spectre family that is additionally self-similar. This can be done via a standard procedure with shape changes that build on tools from algebraic topology [see Anderson & Putnam (1998 ▸), Clark & Sadun (2006 ▸), Sadun (2008 ▸) for general background, and Baake *et al.* (2025*d* ▸) for a detailed treatment of the Hat tiling]. This procedure yields the so-called CAP tiling (for the Hat) and CASPr tiling (for the Spectre). These tilings share the same combinatorics as the clusters of Hats/Spectres, and they are both tilings with prototiles of more than one geometric shape. Despite this, they still describe the monotile tilings in the sense that their tiling spaces are topologically conjugate with respect to the translation action of 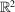.

For the control points of both tilings, CAP and CASPr, we have equations similar to equation (1). Here, the displacement matrix for the CAP tiling is of dimension 24 (four non-congruent prototiles, each in six orientations), whereas for the CASPr, the dimension is 30 (five translational prototiles, each in six orientations).

The following theorem summarizes the model set description of the control points of the CAP and the Hat tilings. For the Spectre tiling, an analogous description is possible, and we refer the reader to Mazáč (2025*a* ▸) for details.

**Figure 4:**
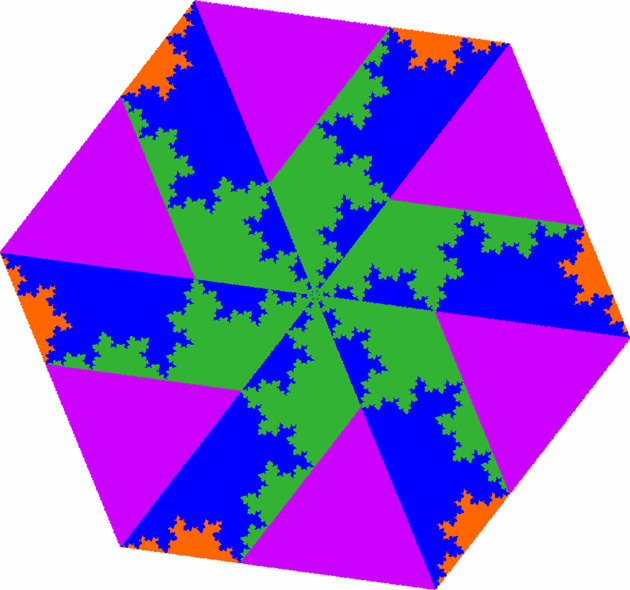
The window for the control points of the CAP tiling. The four different colours correspond to the four different shapes of prototiles (and hence four classes of control points). For more details, see Baake *et al.* (2025*d* ▸), Mazáč (2025*a* ▸).

Theorem 3.1 The control points of the CAP tiling, 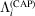, grouped according to the 24 prototiles, are model sets with windows from Fig. 4 ▸ that arise from a Euclidean cut-and-project scheme[Chem scheme2]
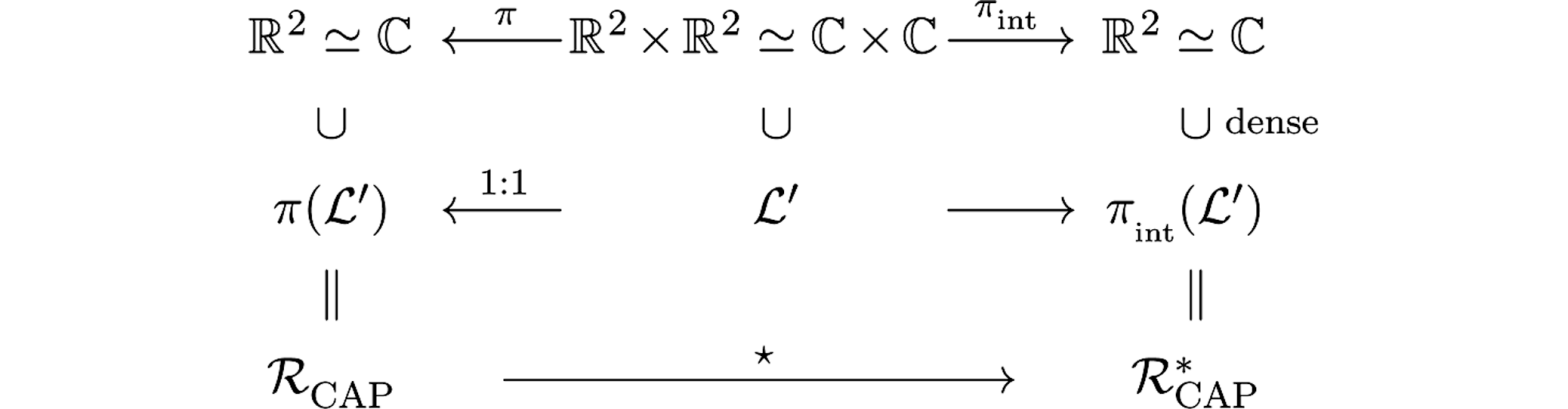
with 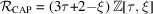 and the star map given by 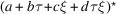 = 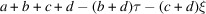

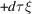
, with 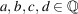, where ξ stands for a primitive sixth root of unity.

The set of control points of the Hat tiling, 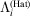, are reprojected model sets, 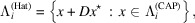
with
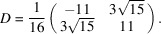

The diffraction theory of regular model sets is well known. For the fully Euclidean setting, the diffraction measure is pure point, and the Bragg peaks are supported by the Fourier module 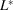, which is the projection of the dual lattice 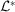 to the physical space. For the diffraction intensities *I*, the covariograms of the windows enter via 
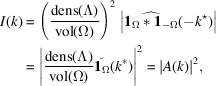
which requires the Fourier transform of a Rauzy fractal. To do so, one has to use the inflation structure again and employ renormalization techniques. For tilings with Euclidean model set description, a method was developed by Baake & Grimm (2020*a* ▸) using a matrix cocycle induced by the original inflation. The method starts with an 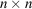 matrix function defined over the internal space, every entry consisting of the inverse Fourier transform of Dirac masses placed at positions given by the 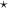-images of the entries of the displacement matrix *T*. For 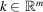 (the internal space), the matrix elements read 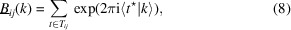
which is denoted by 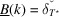 for obvious reasons, and called the *internal Fourier matrix*.

It induces a matrix cocycle, 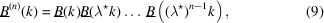
which allows one to consider a matrix function 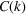 defined by 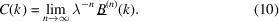
The matrix function 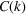 is well defined and continuous, as the sequence 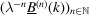 converges compactly on 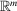 (Baake & Grimm, 2020*a* ▸, Thm. 4.6). Importantly, the convergence of (10) is exponentially fast, which makes it effectively computable to any desired precision. As the matrix 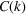 is of rank at most 1, one can rewrite it in Dirac notation as 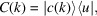
with the properly normalized left Perron–Frobenius (PF) eigenvector 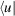 of the inflation matrix 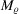. It turns out that the vector of functions 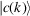 has components 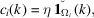
for some constant 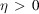, which can be determined explicitly, thus providing the desired quantities [see Baake & Grimm (2020*a* ▸, Sect. 4), Baake & Grimm (2020*b* ▸) for details].

**Figure 5:**
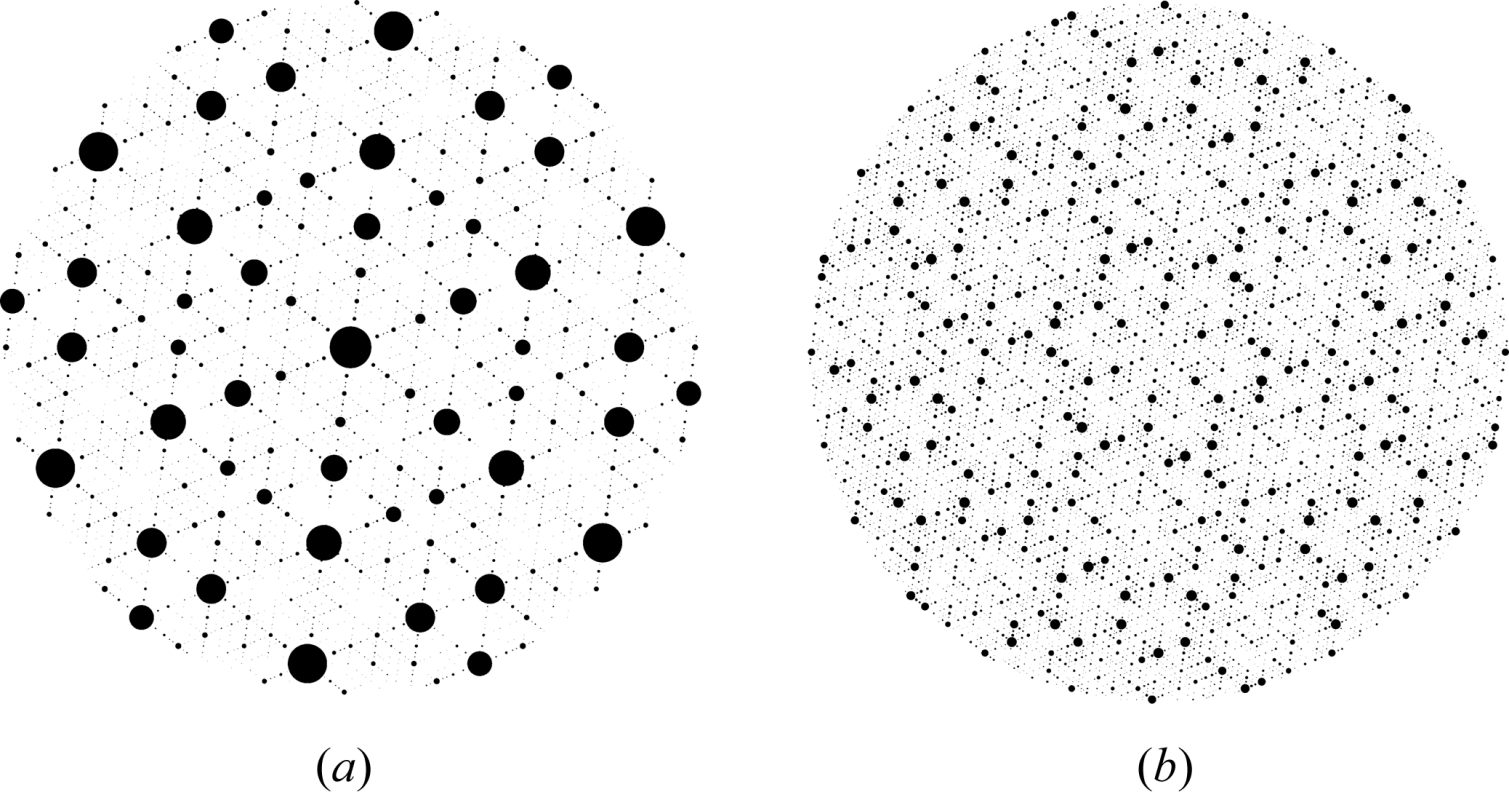
Diffraction pattern of the CAP tiling in the centred ball of radius 0.6. Panel (*a*) shows the case when all control points have equal weights, whereas panel (*b*) shows the diffraction for weights chosen so that the central peak vanishes. See Baake *et al.* (2025*b* ▸), Mazáč (2025*a* ▸) for a detailed discussion of the diffraction pattern.

The Bragg peaks of the CAP tiling are located at the points from the Fourier module 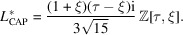
To compute the intensities, one considers the internal Fourier matrix of dimension 24 and approximately 15 iterations of the cocycle, which is sufficient to get precision up to ten decimal places (Mazáč, 2025*a* ▸). Fig. 5 ▸ presents the diffraction image for the CAP tiling.

**Figure 6:**
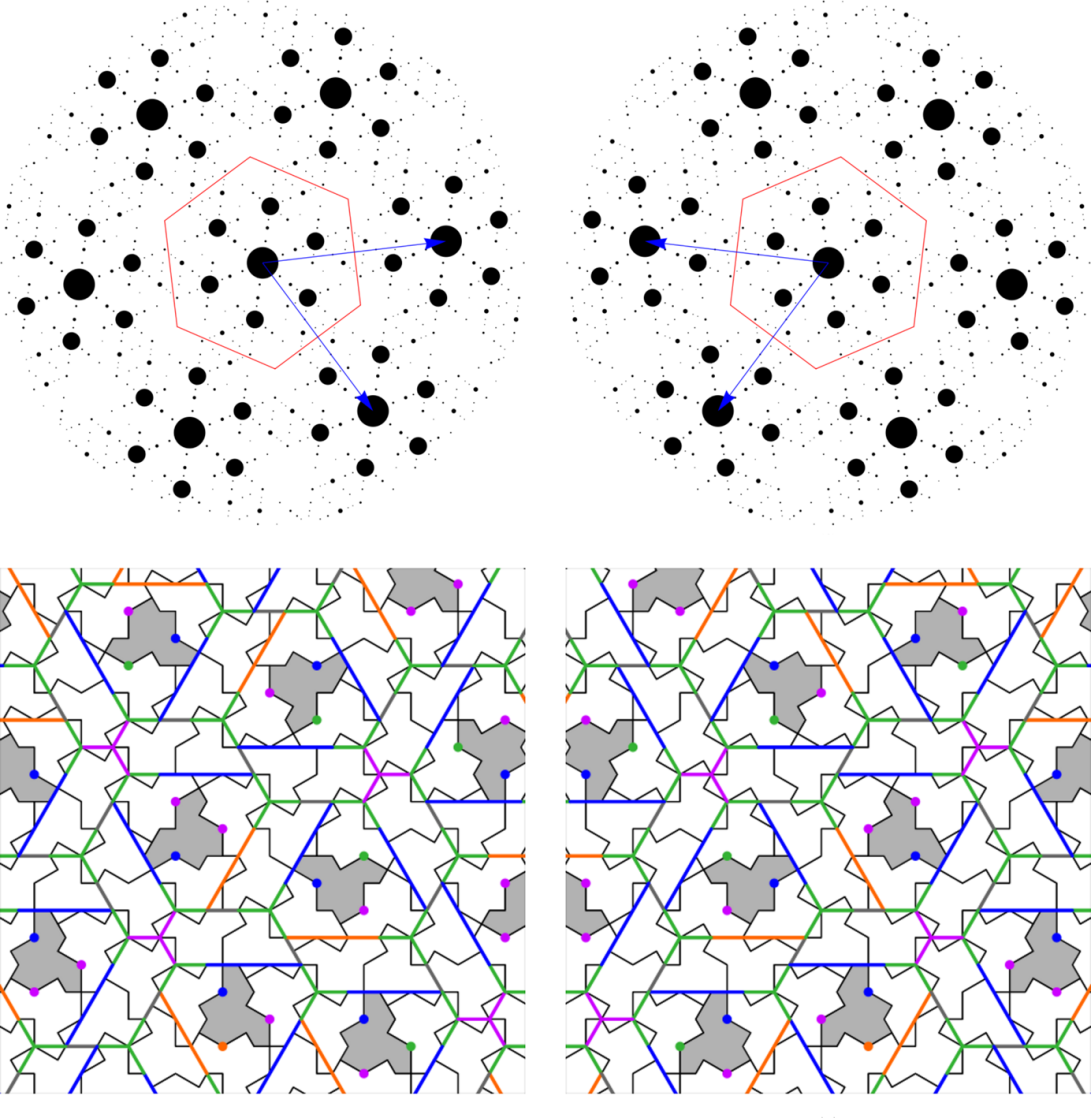
Diffraction of the Hat tiling with equal weights for the two LI classes of the Hat tiling (with indicated control points), each depicted under the corresponding diffraction image. Both pictures display a lattice-periodic structure, with lattice 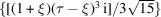

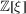
 for the left one and its mirror image for the right one. For further details, we refer the reader to Baake *et al.* (2025*b* ▸) or to Mazáč (2025*a* ▸), where a continuous transformation from the CAP to Hat tiling on the level of the diffraction is depicted.

To obtain the same picture for the Hat tiling, we only need to compute the diffraction intensities. Since the Hat tiling is a reprojection of the CAP tiling, this has to be reflected in the diffraction picture. Indeed, the positions of Bragg peaks remain unchanged [as the two tiling dynamical systems are topologically conjugate (Baake *et al.*, 2025*b* ▸)] and the diffraction intensities 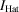 for the Hat tiling are given by the same intensity function 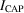 as before, only evaluated at a modified position, namely, for any 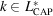, 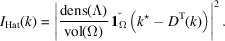
The control points of the Hat tiling are a subset of a hexagonal lattice, which implies that, although the Hat tiling itself is aperiodic, it has a periodic diffraction pattern, with the lattice of periods being the dual lattice of the underlying hexagonal lattice [which is true for any lattice subset (Baake & Grimm, 2013 ▸, Thm. 10.3)]. The aperiodicity of the tiling is present in the fundamental domain, as our figures illustrate. We can use the diffraction image to distinguish the two LI classes (Fig. 6 ▸).

**Figure 7:**
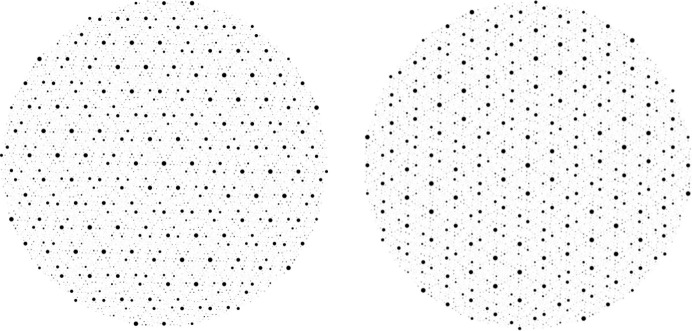
Diffraction of the Spectre tiling with equal weights for the corresponding two LI classes of the Spectre tiling (with indicated control points), each depicted under the corresponding diffraction image (Bragg peaks in a ball of radius 0.5). For further details, we refer the reader to Baake *et al.* (2025*b* ▸), Mazáč (2025*a* ▸).

The same applies to the Spectre tilings, whose diffraction pictures are shown in Fig. 7 ▸. We note that, in this case, we can also use diffraction to detect the two LI classes and to demonstrate the absence of 12-fold symmetry.

Although the approach is quite general, it will not work for the Taylor–Socolar monotile, even though it can be described as a model set. Unfortunately, one needs a non-Euclidean internal space, for which the Fourier cocycle method and the deformation arguments have not yet been established. On the other hand, as this monotile tiling is limit-periodic, a sufficiently good approximation of the diffraction image can be obtained from its periodic approximants, which is a strategy that always more or less works for limit-periodic tilings.

## 4. Orbit separation dimension as topological invariant

The *orbit separation dimension* (OSD) is an invariant for dynamical systems with topological pure-point spectrum, which was introduced under the name *amorphic complexity* (Fuhrmann *et al.*, 2016 ▸; Fuhrmann & Gröger, 2020 ▸). The intention was to have a tool to distinguish between different levels of complexity for systems with zero entropy. Baake *et al.* (2025*a* ▸) showed that the OSD is practically computable for primitive inflation tilings, and indeed is powerful enough to distinguish many different tiling dynamical systems.

In this section, we argue that the OSD is actually closely related to autocorrelation and diffraction. Baake *et al.* (2025*a* ▸) showed that the OSD is equal to the Hausdorff dimension of the discrete hull 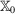 of the tiling (consisting of those tilings that have a control point at the origin), measured with a suitable pseudo-metric *D*. In this pseudo-metric, the distance between two tilings is given by the volume fraction covered by tiles which do not occur in both tilings, that is, the volume fraction of the region where the two tilings do not agree. The complement is the region covered by coincident tiles. With suitable weights for the different tile types, the latter is equal to the total correlation between the two tilings, that is, the fraction of control points which agree in position and type between the two tilings. Hence, the pseudo-metric *D* is equivalent to the autocorrelation pseudo-metric.

For inflation tilings, the metric space 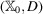 [or rather its projection to the *maximal equicontinuous factor* (MEF), on which *D* is a proper metric] can be constructed as the fixed point of an IFS, and its dimension is determined by the contraction rate of the distance 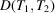 between two tilings under iterated inflation. In particular, for a tiling *T* and its translate 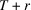 by a return vector *r*, their distance under iterated inflation 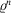 becomes 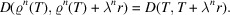
For projection tilings, one finds 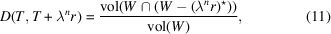
where *W* is the window of *T* and 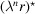 is the 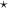-image of 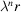. For a Pisot inflation factor, 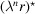 is contracting and converges to zero, so that the two windows converge to each other. Solomyak has shown that an inflation tiling has pure-point spectrum, and hence is a projection tiling, if and only if 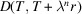 converges to zero for every return vector *r* (Solomyak, 1997 ▸; Solomyak, 1999 ▸).

As we have remarked above, the OSD is determined by the contraction rate of 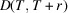 under inflation. This rate can be determined as follows. The superposition of the tilings *T* and 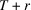 is dissected into so-called overlaps, pairs of tiles (one from each tiling) whose supports have an overlap with non-empty interior. There are coincidence overlaps between identical tiles, and discrepancy overlaps between non-coincident tiles. Up to translation, there are finitely many overlap types, and the inflation on tiles induces an inflation on overlaps. According to Solomyak, for tilings with pure-point spectrum, every discrepancy overlap eventually produces a coincidence, so that the discrepancy region strictly shrinks under inflation. The contraction rate is given by the growth of the number of discrepancies (controlled by the leading eigenvalue 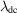 of the discrepancy part of the overlap inflation), divided by the growth of the number of all overlaps. For a tiling of 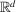, we get (Baake *et al.*, 2025*a* ▸) 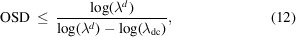
where in many cases one can actually show equality.

The same can also be obtained from (11), which relates the OSD to the rate of convergence of the sequence 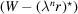 to *W*. As one can imagine, this depends on how complicated the boundary of *W* is. Indeed, also the window boundary is the fixed point of an IFS, which is closely related to the overlap inflation (see Mazáč, 2025*a* ▸). In nice cases (Euclidean internal space of dimension 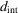, with isotropic contraction under inflation) one finds that 
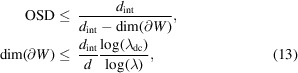
where, again, one can show equality in many cases.

We see now that the same eigenvalue 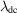 governs, together with the inflation factor λ, the convergence of the correlation between *T* and 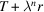 to a limiting value, the contraction of the discrepancy region between *T* and 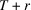 under inflation, the dimensions of the discrete hull 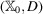 and the window boundary 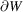, and presumably the convergence of the Fourier–Bohr coefficients with increasing internal space component of the *k* vector (which depends on the dimension of the window boundary).

**Figure 8:**
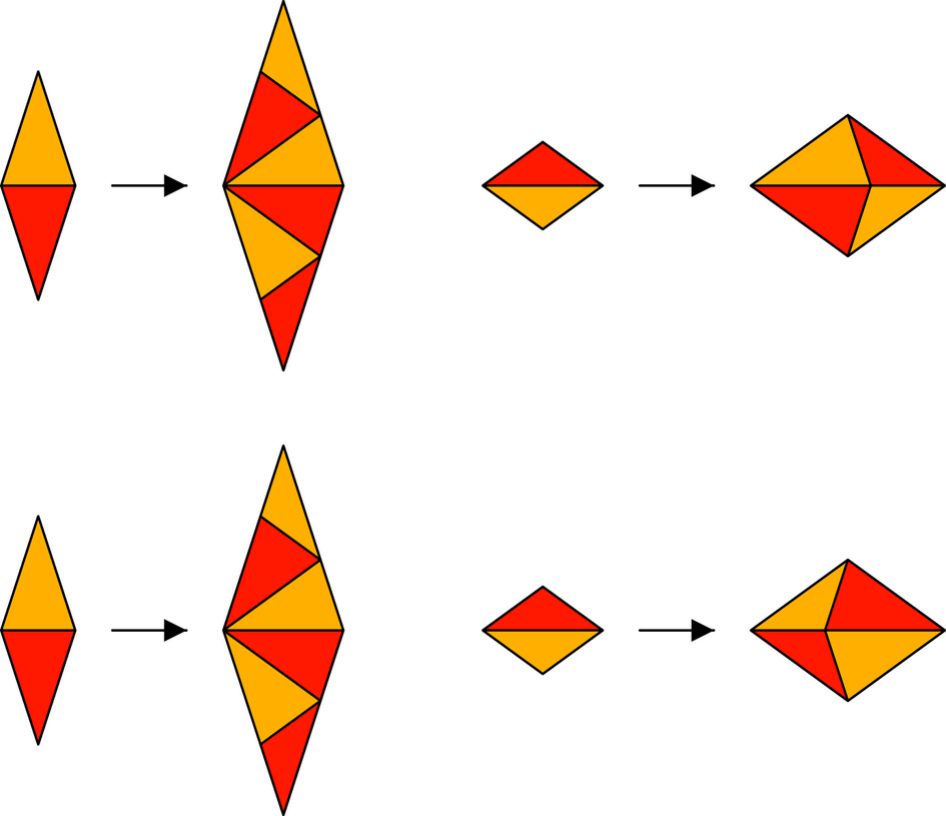
Inflation of the Penrose tiling (top) and the pentagonal tiling (bottom).

**Figure 9:**
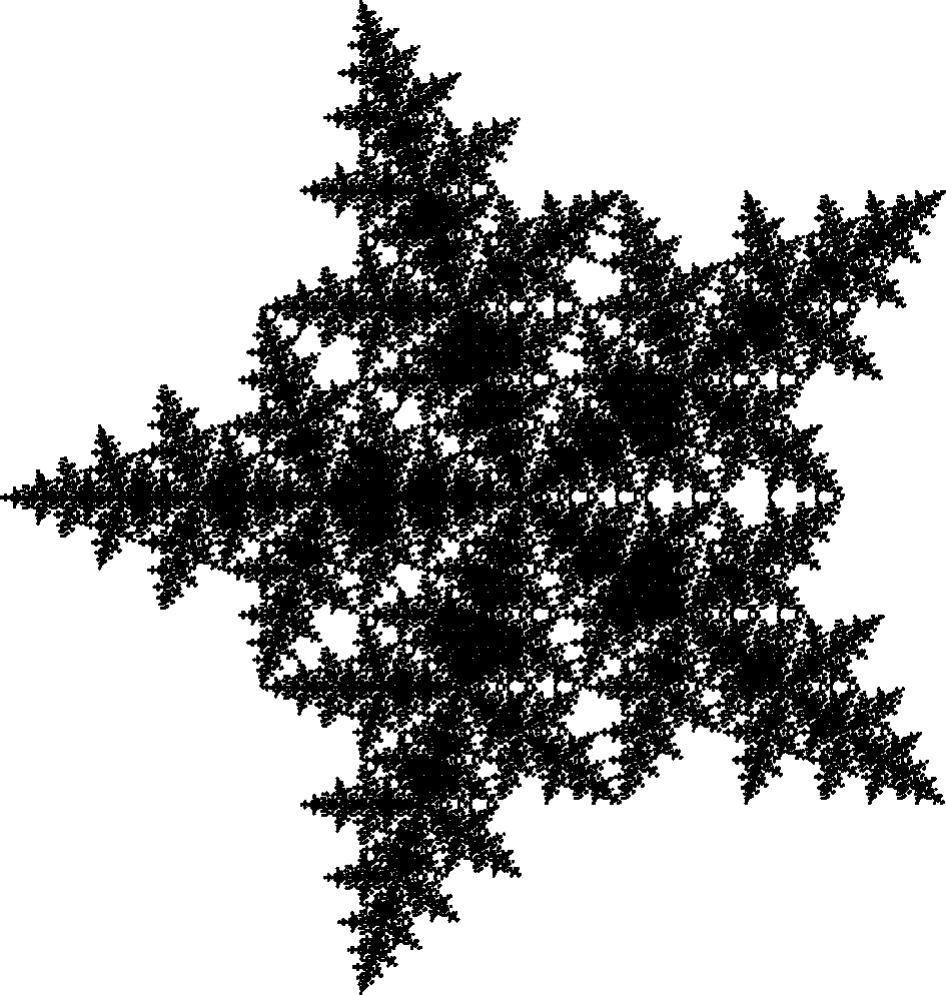
The window of the pentagonal tiling, defined by the rule from Fig. 8 ▸ (bottom).

As an example, we mention the Hat tiling, whose internal window boundary (see Fig. 4 ▸) has (parts of) Hausdorff dimension 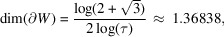
with τ as above. This relates to the OSD as 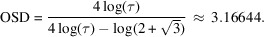
As a second example, we compare a Penrose tiling (by Robinson triangles) with a closely related pentagonal tiling (compare Fig. 8 ▸). For the Penrose tiling, all four tile types occur in ten orientations each, whereas for the pentagonal tiling, there are only five orientations per tile. The Penrose tiling has 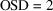, and it is MLD to a pattern with a regular decagon as its window, whereas the pentagonal tiling has 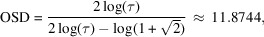
with a window boundary dimension 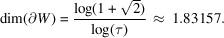
Its window is the pentagonal fractal snowflake shown in Fig. 9 ▸.

## 5. Beyond pure-point diffraction – how to exclude absolutely continuous contributions to the spectrum

The previous sections describe the renormalization properties satisfied by objects related to the pure-point part of the spectrum (covariogram, window, intensities of the Bragg peaks). Apart from these, the inflation structure also induces an exact renormalization at the level of the *absolutely continuous* component of the diffraction, which is mathematically described by a function 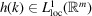 called its *Radon–Nikodym density*.

One can write 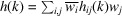, where 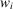 is the (complex) weight assigned to a control point of type *i*. The vector 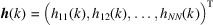
then satisfies the equation 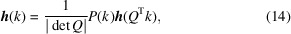
where 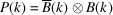, with 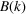 being the Fourier matrix in *physical* space [as opposed to the internal Fourier matrix given in equation (8)]; see Baake *et al.* (2019*b* ▸) for details. The main difference is that the entries are trigonometric polynomials in 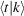 instead of 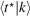. Here, the map 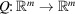 is the geometric expansion map associated to ϱ.

One can invoke a dimensional reduction argument to obtain an equation involving only 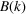 instead of 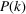. This leads to the Fourier cocycle 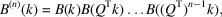
which is the analogue of equation (9) in physical space. For one-dimensional inflation rules, the inflation factor 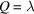 coincides with linear scaling. In higher dimensions, the effective inflation factor is given via 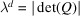.

The maximal *Lyapunov exponent*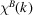 of this cocycle is given by 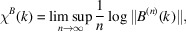
where the choice of norm is clearly immaterial. This quantity provides access to the nature of the absolutely continuous component, which the next result sums up; see Baake *et al.* (2019*b* ▸), Mañibo (2019 ▸) for details.

Theorem 5.1 (Baake et al., 2019b, Thm. 5.7) Let ϱ be a primitive inflation in 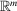 on finitely many prototiles, with inflation map *Q*. If there exists 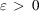 such that 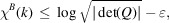
for a.e. 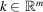, the diffraction of any (weighted) point set constructed from ϱ has no absolutely continuous component.

An additional non-degeneracy condition is assumed in the preceding theorem, namely 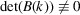. In the case where 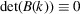, it is sometimes possible to identify the subspaces where 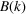 is non-invertible and restrict the analysis to the complement.

The basic principle behind the proof is simple: if 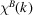 is bounded away from 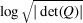, one can show that 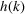 grows exponentially along a 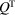-invariant subset of 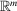 of positive Lebesgue measure. On the other hand, the absolutely continuous component inherits the translation-boundedness of the entire diffraction measure. The only scenario where these two behaviours are compatible is when 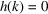, for a.e. 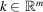.

Theorem 5.1[Statement theorem5.1] and the objects involved are powerful in many respects. This criterion holds in *any* dimension and is applicable to systems with mixed spectral type (that is, those with non-trivial pure-point component). Moreover, it is able to treat systems with non-Pisot λ, all of which do not admit a covering model set, and hence do not possess a (reasonable) description via the window *W* in internal space.

In its current form, it is also applicable to certain systems with infinite local complexity, such as tilings that are not edge-to-edge, provided there exist only finitely many prototiles up to translation.

We now present a procedure how to implement Theorem 5.1[Statement theorem5.1] to check for singularity of the diffraction for a given example ϱ; see Baake *et al.* (2019*a* ▸), Baake *et al.* (2019*b* ▸), Baake *et al.* (2018 ▸), Mañibo (2019 ▸) for fully worked-out examples.

(1) From the inflation ϱ, compute the displacement matrix *T*.

(2) From the displacement matrix *T*, build the Fourier matrix 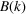. If the non-degeneracy condition holds for 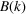, one can directly build the Fourier cocycle 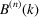.

As an example, 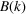 for the Godrèche–Lançon–Billard (GLB) inflation rule (Lançon & Billard, 1988 ▸) is a 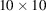 matrix valued function. Two of the entries are given below: 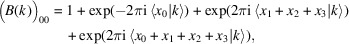

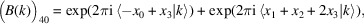
for 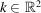 (Fig. 10 ▸).

(3) Consider 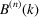 as a section of a periodic function 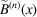, 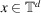 for some 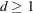 (either via cyclotomic extensions, or via the action of the adjacency matrix of the minimal polynomial of λ) (see Mañibo, 2019 ▸, Sec. 5.3).

(4) Compute Kingman-type bounds 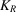 for the cocycle 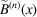, which are of the form 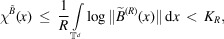
for 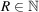 and for a.e. 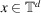. This extends to bounds for 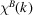, which is justified by sampling results for Stepanov almost periodic functions along equidistributed sequences (Baake *et al.*, 2017 ▸).

(5) Note that Kingman-type bounds are obtained from the integral of 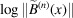 over 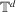. Approximating such integrals can be done via numerical integration.

(6) If the obtained bound 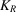 is less than 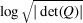, for some 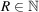, there is no absolutely continuous diffraction.

For the GLB inflation, an upper bound 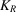 for 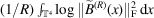 crosses the threshold 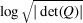 for 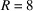, where 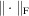 is the Frobenius norm (see Baake *et al.*, 2019*b* ▸; Mañibo, 2019 ▸).

In fact, whenever Theorem 5.1[Statement theorem5.1] holds, the bounds for the Lyapunov exponent do not only provide the mere absence of absolutely continuous diffraction, but also bounds for the lower local dimension of the corresponding spectral measures (see Bufetov & Solomyak, 2018 ▸; Bufetov *et al.*, 2025 ▸; Solomyak & Treviño, 2024 ▸). This unlocks the route towards *quantitative weak mixing*, where exponential decay rates for correlation measures (via the Hölder exponents) can be derived from the bounds for Lyapunov exponents. These are robust bounds in the sense that they are invariant under *admissible deformations*, which are shape changes on the prototiles that do not alter the combinatorics of the tiling (*e.g.* adjacency of faces, connectedness of vertices *etc*.) (see Solomyak & Treviño, 2024 ▸).

It would be interesting to apply the procedure described above to larger classes of non-Pisot systems. For example, the parametrized family of square–triangle tilings presented by Say-awen & Coates (2025 ▸) (originally motivated by tiling models for soft-matter quasicrystals) admits a subclass with non-Pisot inflation. Confirming singularity for this class of tilings would be a good first step in describing and visualizing their spectrum.

## Figures and Tables

**Figure 10:**
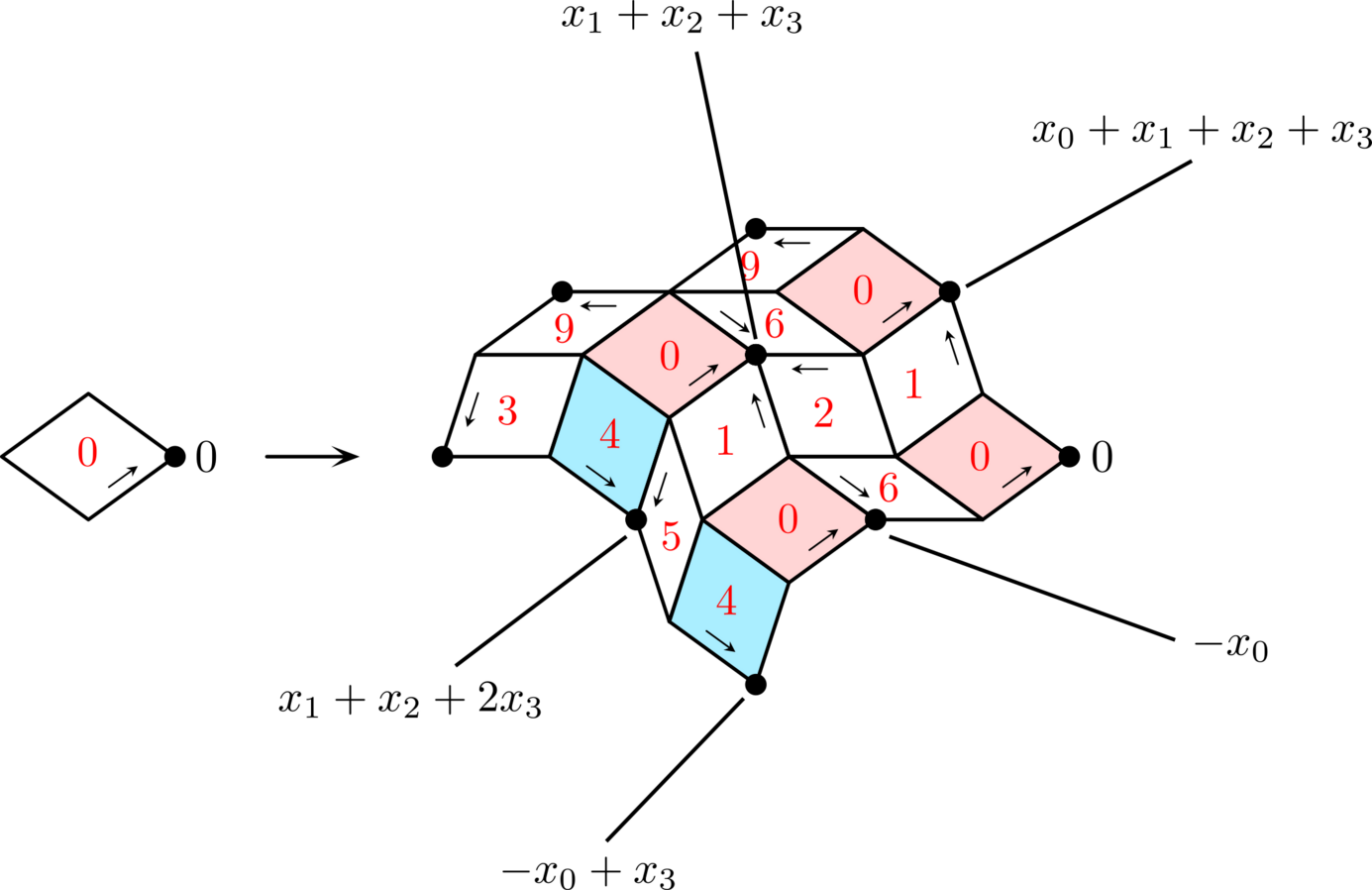
The level-1 supertile of type 0 for the Godrèche–Lançon–Billard (GLB) inflation rule. The location of the type-0 (pink) and type-4 (blue) tiles are labelled, where one has 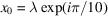, 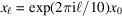 (for 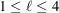) and 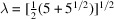; see Baake *et al.* (2019*b* ▸), Mañibo (2019 ▸) for a complete account.

**Table 1:** The self-consistent part of the renormalization equations for the tiling given by ϱ, with the natural tile lengths given by the left PF eigenvector Distances that are not possible, *i.e.* ones for which all pair correlations evaluate to 0 due to the tile geometry, are omitted. The column corresponding to 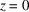 contains the relative tile frequencies from the (statistically normalized) right PF eigenvector of 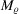.

*z*	0	1	2	λ	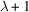	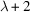	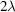	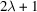
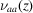	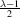	0	0	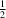	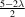	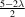	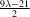	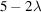
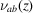	0	0	0	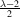	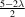	0	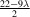	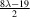
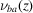	0	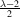	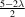	0	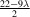	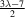	0	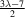
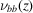	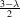	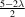	0	0	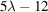	0	0	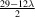

## Data Availability

Data are available upon request to the corresponding author. For further information, please consult the *arXiv* version.

## References

[bb1] Akiyama, S., Barge, M, Berthé, V., Lee, J.-Y. & Siegel, A. (2015). *Mathematics of Aperiodic Order*, edited by J. Kellendonk *et al.*, pp. 33–72. Basel: Birkhäuser.

[bb2] Anderson, J. E. & Putnam, I. F. (1998). *Ergod. Th. Dyn. Sys.* **18**, 509–537.

[bb3] Baake, M., Frank, N. P., Grimm, U. & Robinson, E. A. Jr (2019*a*). *Stud. Math.* **247**, 109–154.

[bb4] Baake, M. & Gähler, F. (2016). *Topology and its Applications* **205**, 4–27.

[bb5] Baake, M., Gähler, F. & Gohlke, P. (2025*a*). *Ergod. Th. Dyn. Sys.* **45**, 2992–3020.

[bb6] Baake, M., Gähler, F. & Mañibo, N. (2019*b*). *Commun. Math. Phys.* **370**, 591–635.

[bb7] Baake, M., Gähler, F., Mazáč, J. & Mitchell, A. (2025*b*). *J. Math. Phys.* **66**, 092707, 1–26.

[bb8] Baake, M., Gähler, F., Mazáč, J. & Sadun, L. (2025*c*). *Discrete Comput. Geom.*https://doi.org/10.1007/s00454-025-00756-z.

[bb9] Baake, M., Gähler, F. & Sadun, L. (2025*d*). *Isr. J. Math.* **270**, 449–485.

[bb10] Baake, M., Gorodetski, A. & Mazáč, J. (2024). *Lett. Math. Phys.* **114**, 101.

[bb11] Baake, M. & Grimm, U. (2013). *Aperiodic Order*, Vol. 1, *A Mathematical Invitation*. Cambridge University Press.

[bb12] Baake, M. & Grimm, U. (2020*a*). *Doc. Math.* **25**, 2303–2337.

[bb13] Baake, M. & Grimm, U. (2020*b*). *J. Phys. Conf. Ser.* **1458**, 012006.

[bb14] Baake, M., Grimm, U. & Mañibo, U. (2018). *Lett. Math. Phys.* **108**, 1783–1805.

[bb15] Baake, M., Haynes, A. & Lenz, D. (2017). *Aperiodic Order*, Vol. 2, *Crystallography and Almost Periodicity*, edited by M. Baake and U. Grimm, pp. 343–362. Cambridge University Press, Cambridge.

[bb16] Baake, M., Klick, A. & Mazáč, J. (2025*e*). *arXiv*:2502.20487, *J. Austral. Math. Soc*. In the press.

[bb18] Bufetov, A., Marshall–Maldonado, J. & Solomyak, B. (2025). *J. London Math. Soc.* **111**, e70136.

[bb17] Bufetov, A. & Solomyak, B. (2018). *Journal d’Analyse Mathématique* **141**, 165–205.

[bb19] Clark, A. & Sadun, L. (2006). *Ergod. Th. Dyn. Sys.* **26**, 69–86.

[bb20] Fuhrmann, G. & Gröger, M. (2020). *Math. Z.* **295**, 1385–1404.

[bb21] Fuhrmann, G., Gröger, M. & Jäger, T. (2016). *Nonlinearity* **29**, 528–565.

[bb22] Hollander, M. & Solomyak, B. (2003). *Ergod. Th. Dyn. Sys.* **23**, 533–540.

[bb23] Klick, A. (2024). Master thesis, University of Bielefeld, Germany.

[bb24] Lagarias, J. C. & Wang, Y. (1996). *J. London Math. Soc.* **54**, 161–179.

[bb25] Lançon, F. & Billard, L. (1988). *J. Phys. Fr.* **49**, 249–256.

[bb26] Lee, J.-Y., Moody, R. V. & Solomyak, B. (2002). *Ann. H. Poincaré* **3**, 1003–1013.

[bb27] Mañibo, N. (2019). PhD thesis, University of Bielefeld, Germany. urn:nbn:de:0070-pub-29359727.

[bb28] Mazáč, J. (2025*a*). PhD Thesis, University of Bielefeld, Germany. urn:nbn:de:0070-pub-30062996.

[bb29] Mazáč, J. (2025*b*). *arXiv*:2507.07753.

[bb30] Moody, R. V. (2002). *Can. Math. Bull.* **45**, 123–130.

[bb31] Sadun, L. (2008). *Topology of Tiling Spaces*. American Mathematical Society, Providence, RI, USA.

[bb32] Say-awen, A. L. & Coates, S. (2026). *Acta Cryst.* A**82**, 179–190. 10.1107/S2053273326002767 41958281

[bb33] Siegel, A. & Thuswaldner, J. (2009).*Topological Properties of Rauzy Fractals*. Sociéte Mathématique de France, Paris, France.

[bb34] Sing, B. (2007). PhD thesis, University of Bielefeld, Germany. urn:nbn:de:hbz:361-11555.

[bb35] Smith, D., Myers, J. S., Kaplan, C. S. & Goodman-Strauss, C. (2024*a*). *Combin. Theory* **4**, 6.

[bb36] Smith, D., Myers, J. S., Kaplan, C. S. & Goodman-Strauss, C. (2024*b*). *Comb. Theory* **4**, 13.

[bb37] Solomyak, B. (1997). *Ergod. Th. Dyn. Sys.* **17**, 695–738.

[bb50] Solomyak, B. (1999). *Ergod. Th. Dyn. Sys.* **19**, 1685.

[bb38] Solomyak, B. & Treviño, R. (2024). *Ergod. Th. Dyn. Sys.* **44**, 1629–1672.

[bb39] Vince, A. (2000). *Directions in Mathematical Quasicrystals*, edited by M. Baake and R. V. Moody, pp. 329–370. Fields Institute Monographs, Vol. 13, American Mathematical Society, Providence, RI, USA.

